# Effect of Serum 25 Hydroxy Vitamin D Level on Isotretinoin-Induced Musculoskeletal Symptoms: A Cross-Sectional Study

**DOI:** 10.1038/s41598-020-59167-0

**Published:** 2020-02-10

**Authors:** Cevriye Mülkoğlu, Nermin Karaosmanoğlu

**Affiliations:** 1 Department of Physical Medicine and Rehabilitation, Health Sciences University Ankara Training and Research Hospital, Ankara, Turkey; 2 Department of Dermatology, Health Sciences University Ankara Training and Research Hospital, Ankara, Turkey

**Keywords:** Medical research, Rheumatology

## Abstract

Isotretinoin (ISO) is a drug which is used for the treatment of severe and refractory acne vulgaris (AV), over the last few decades. The drug has various musculoskeletal side effects. The aim of this study was to investigate relationship between serum 25 hydroxy (OH) vitamin D levels and the ISO-induced musculoskeletal side effects in patients with AV. We included 87 patients receiving ISO and had musculoskeletal symptoms as adverse effect (AE) group. Another 90 patients receiving ISO for AV and had any musculoskeletal complaints were recruited as control (C) group. Locomotor system examination of the patients was performed by the same clinician. Serum 25 OH vitamin D levels of the all participants were measured. Patients in the AE group were divided into three subgroups by serum 25 OH vitamin D levels. Patients with serum 25 OH vitamin D level lower than 10 ng/ml was classified as Group I, the ones between 10–20 ng/ml as Group II and those higher than 20 ng/ml were classified as Group III. AE and C groups were similar in terms of age and sex (p > 0.05). There was no statistically significant difference in the mean serum vitamin D levels between two groups (p = 0.17). Also, there was no significant difference in number of arthralgia (p = 0.30), myalgia (p = 0.29), low back pain (p = 0.10) and sacroiliitis (p = 0.17) between three subgroups in AE group. In addition, we found no statistically significant correlation between the serum vitamin D levels and age, cumulative dose of ISO, arthralgia, myalgia and sacroiliitis parameters in AE group (p > 0.05). Serum 25 OH vitamin D levels between the AE and C groups were similar. We also found that no significant difference in musculoskeletal adverse events between AE subgroups. Therefore, it can be concluded that vitamin D deficiency has no effect on the musculoskeletal adverse events in patients receiving ISO.

## Introduction

Acne vulgaris (AV) is the most common chronic inflammatory skin disease among the adolescents. It affects more than 80% of teenagers. It is estimated that 9.4% of the world population is affected by AV. AV may cause psychological and social disorders related to anxiety due to facial scarring and reduced self esteem, these are significant burden on the affected patients.

Isotretinoin (ISO) is 13-cis-retinoic acid, a synthetic vitamin A analogue and the most effective drug in treatment of recalcitrant AV for more than three decades^1–5^. The usual starting dose is 1–2 mg/kg/day to achieve a cumulative dose of 120–150 mg/kg over 4–5 months.

ISO has a wide spectrum of adverse effects on multiorgan systems, including reproductive, cutaneous, ocular, neurological, musculoskeletal, and hepatic. It may cause teratogenicity, hepatitis, dryness of skin, psychological effects and cerebral ischemia. Most of these adverse effects induced by ISO are predictable, dose-dependent and manageable. More than half of all adverse events associated with ISO are mucocutaneous and occur especially in the first weeks of treatment^6–8^. The rheumatic side effects are the most common one which are musculoskeletal pains and arthralgia, occuring in 16% patients receiving ISO. Mild, transient myalgias and arthralgias are very common and do not require cessation of the drug^9,10^. The other musculoskeletal side effects of ISO are calcification of tendon and ligaments, diffuse idiopathic skeletal hyperostosis (DISH syndrome), elevated creatine phosphokinase and cramps^10^. There are also several case reports on ISO-induced sacroiliitis in the literature, mostly in recent years^11–16^.

The association ISO and vitamin D levels was evaluated by several studies in the literature^17–19^. However, the relationship between ISO-induced musculoskeletal side effects in AV patients and serum vitamin D levels has not been investigated until now. To the best our knowledge, this is the first study to evaluate relationship between the serum vitamin D levels and ISO-induced musculoskeletal adverse effects.

## Methods

### Study design

This study was designed as a cross-sectional controlled, single center study at Health Sciences University Ankara Training and Research Hospital, Departments of Physical Medicine- Rehabilitation and Dermatology, between April 2019 and September 2019. The study protocol of the study was approved by institutional clinical trials ethics committee with the number of 74.765,13.03.2019 and registered on ClinicalTrials.gov (NCT04204304). The patients were informed about the study and written consent was obtained from all participants. The study was performed in accordance with the ethical standards specified in the 1964 Declaration of Helsinki and its later amendments.

### Eligibility

In this study, 87 patients receiving ISO for AV with musculoskeletal adverse effects were classified as AE group. Age- and sex-matched 90 consecutive patients receiving ISO without any musculoskeletal symptoms were classified as C group. The patients over 18 years of age and receiving ISO for at least one month were included the study.

ISO was started with the dose of 0.5–1 mg/kg/day and continued to achieve a total cumulative dose of 120 mg/kg over 6 months.

The ones using vitamin D and/or calcium supplements for the last three months were not included. Patients who had renal, gastrointestinal, skeletal, psychiatric, hematological, endocrine disorders related with thyroid and bone metabolism, also, patients using drugs such as diuretics, multivitamins, anticonvulsants, glucocorticoids, erythromycin, estrogen compound pills, alcohol, vitamin D and/or calcium preparations in the last 3 months, patients with malignancy, chronic liver and kidney failure, history of psoralen and ultraviolet A (PUVA) and women expecting pregnancy were excluded from the study.

### Assessments during the study

**Figure 1:**
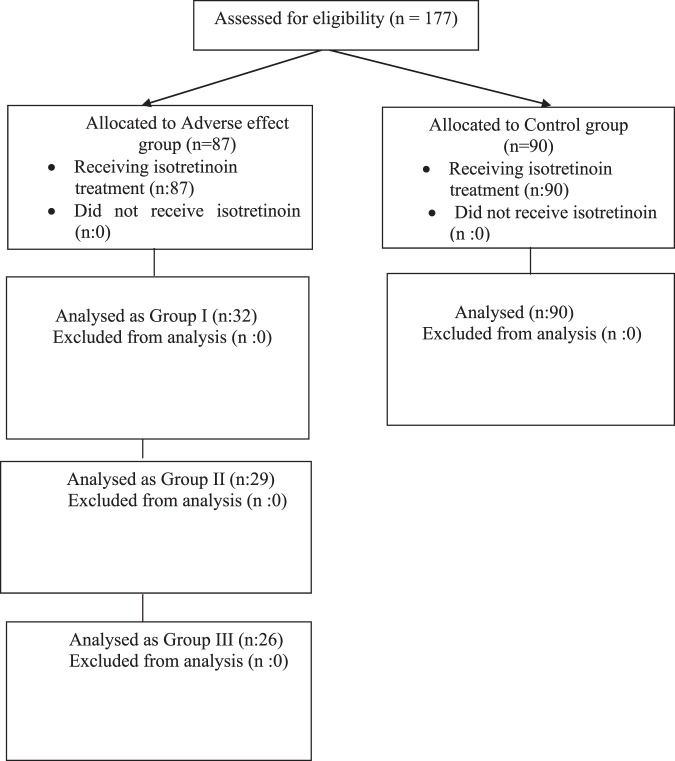
The flowchart of the participants in the study.

The demographic and clinical characteristics of the participants were noted. Age and sex of the participants, treatment duration and adverse events induced with ISO were recorded. The cumulative ISO dose was calculated. The study flowchart is shown in Fig. 1.

Physical examination of the patients was performed by the same experienced clinician. The musculoskeletal symptoms including arthralgia, low back pain, calcification of tendon and ligaments, polyneuropathy, DISH syndrome, myalgia, cramps and sacroiliitis were assessed. It was queried that whether musculoskeletal symptoms have occurred after ISO treatment. Inflammatory low back pain was evaluated by Assessment of Spondyloarthritis International Society (ASAS) criteria. The ASAS criteria consist of onset at the age of 40, insidious onset, improvement with exercise, no improvement with rest and nocturnal pain (improving with rising). Inflammatory low back pain is diagnosed in the presence of these four criteria. Patients with such criteria were assessed by a clinician in the physical medicine and rehabilitation department. Sacroiliac joint radiography was requested in patients meeting the ASAS criteria. Magnetic resonance imaging of the sacroiliac joints was used to diagnose sacroiliitis in suspected patients. The serum 25 OH vitamin D levels were measured by radioimmunoassay for all the participants. These results showed that patients in AE group were divided into three subgroups according to the serum 25 OH vitamin D levels. The patients with serum 25 OH vitamin D level is lower than 10 ng/ml, was classified as Group I, between 10–20 ng/ml, as Group II, higher than 20 ng/ml as Group III.

### Power analysis

The power analysis of the present study was conducted using the software package, G*Power Ver.3.1 (Germany)^20^. The power analysis revealed that this study had 90% power using type I error (α) = 0.05, effect size = 0.5, and a two-sided t test.

### Statistical analysis

Statistical analysis was performed using SPSS version 21.0 (IBM Corp., Armonk, NY, USA). Kolmogorov-Smirnov test was used to evaluate the normality of data distribution. Normal distribution data were expressed as mean (±standard deviation), and data not conforming to normal distribution were expressed as median (interquartile distribution (iqd)). Independent samples t test was used to compare the study groups. Kruskal Wallis test was used to compare cumulative ISO dose between the study groups. The relationship between serum 25 OH vitamin D level and cumulative dose of ISO was calculated by using Spearman’s correlation analysis. Pearson correlation analysis was used to assess the relationship between the serum 25 OH vitamin D level and demographic/clinical characteristics of the patients in AE group. Pearson Chi-square test was used for comparison of the subgroups in terms of arthralgia, myalgia, low back pain and sacroiliitis. p < 0.05 was considered statistically significant for all tests.

### Ethics approval and consent to participate

This study was approved by the Health Sciences University, Ankara Training and Research Hospital, Clinical Trials Ethics Committee (no: 74.765, 13.03.2019). Please see Supplementary information.

### Consent for publication

Informed consent was obtained from all the participants for publication of this study.

## Results

In this study, we evaluated 87 patients with musculoskeletal symptoms related with ISO and 90 patients without any musculoskeletal symptoms in terms of serum 25 OH vitamin D level.

67 of patients in AE group were female and 20 were male. The mean age of the patients in AE group was 20.8 ± 4.0. The C group consisted of 68 females and 22 males. The mean age of the C group was 22.1 ± 4.0 years. There was no significant difference in age and sex between the groups (p = 0.18, p = 0.12, respectively).

The mean serum 25 OH vitamin D level of the patients was 13.5 ± 6.9 ng/ml (range 3.0 to 37.9) in AE group and 12.1 ± 5.6 ng/ml (range 4.0 to 31.5) in C group. There was no statistically significant difference in the serum vitamin D levels between two groups (p = 0.17). The mean duration of ISO treatment was 3.4 ± 1.9 (1–8) months in AE group and 3.7 ± 2.2 (2–7) months in C group. The median cumulative dose of ISO was 2400 (3000) mg for AE group and 2800 (3200) mg for C group. There was no significant difference in cumulative dose of ISO and treatment duration between the groups (p = 0.31, p = 0.43, respectively).

The demographic and clinical characteristics of two groups are presented in Table 1.

**Table 1:** The clinical and demographic characteristics of the participants receiving ISO.

	AE group (n:87)	C group (n:90)	p value
Sex	20 males 67 females	22 males 68 females	0.12
The mean age(years) ± SD	20.8 ± 4.0	22.1 ± 4.0	0.18
The mean treatment duration (months)	3.4 ± 1.9	3.7 ± 2.2	0.43
The median cumulative dose of ISO(iqd)(mg)	2400 (3000)	2800 (3200)	0.31
The mean serum 25 OH vitamin D level (ng/ml) ± SD	13.5 ± 6.9	12.1 ± 5.6	0.17

Independent samples t test was used to compare the study groups. Kruskal Wallis test was used to compare cumulative ISO dose between two groups. n: Number; AE: Adverse effect; C: Control; SD: Standard deviation; ISO: Isotretinoin; iqd: Interquartile distribution. p value < 0,05 was considered as statistically significant.

The patients in AE group were divided into three subgroups according to the serum vitamin D levels. The patients whose serum 25 OH vitamin D level is lower than 10 ng/ml were classified as Group I, between 10–20 ng/ml as Group II and higher than 20 ng/ml as Group III. There were 32 patients in Group I, 29 patients in Group II and 26 patients in Group III. We found no statistically significant difference in age and sex between the subgroups (p = 0,18, p = 0,20, respectively). The mean serum 25 OH vitamin D levels was 7 ± 1.9 ng/ml in Group I, 14.7 ± 2.6 ng/ml in Group II and 26.1 ± 1.6 ng/ml in Group III. There was a significant difference in serum vitamin D levels between the subgroups (p < 0.001). The characteristics of subgroups in AE group are shown in Table 2.

**Table 2:** The characteristics of the subgroups in AE group.

	Group I (n:32)	Group II (n:29)	Group III (n:26)	p value
Sex	24 females 8 males	19 females 10 males	19 females 7 males	0.20
The mean age(years) ± SD	19.8 ± 3.0	21.1 ± 3.5	21.5 ± 4.0	0.18
The mean serum 25 OH vitamin D levels (ng/ml) ± SD	7 ± 1.9	14.7 ± 2.6	26.1 ± 1.6	**<0.001**
Arthralgia	15	13	14	0.30
Myalgia	16	15	16	0.29
Low back pain	19	21	19	0.10
Sacroiliitis	5	4	3	0.17

Pearson Chi-square test was used to compare the characteristics of the patients in the subgroups. n: Number; SD: Standard deviation. p value < 0,05 was considered as statistically significant.

In AE group, 42 (48.3%) patients had arthralgia, 47 (54%) had myalgia, 59 (67.8%) had low back pain and 12 (12.6%) had sacroiliitis. There was no statistically significant difference between the subgroups in terms of arthralgia (p = 0.30), myalgia (p = 0.29), low back pain (p = 0.10) and sacroiliitis (p = 0.17).

We did not find any correlation between the serum 25 OH vitamin D levels and age, cumulative dose of ISO, treatment duration, arthralgia, myalgia, low back pain and sacroiliitis parameters in AE group (all p > 0.05). The results of correlation analysis between the serum vitamin D levels and demographic and clinical characteristics of the patients in AE group are summarized in Table 3.

**Table 3:** Correlation between the serum 25 OH vitamin D levels and demographic and clinical characteristics of the patients in AE group.

	p value
Age (years)	0.09
Cumulative dose of isotretinoin (mg)	0.49
Treatment duration (months)	0.85
Arthralgia	0.45
Myalgia	0.21
Low back pain	0.32
Sacroiliitis	0.18

Spearman and Pearson correlation analysis was used to determine relationship between the serum 25 OH vitamin D level and demographic/clinical characteristics of the patients in AE group. p value < 0,05 was considered as statistically significant.

## Discussion

In this study, we investigated whether there was a relationship between serum vitamin D levels and musculoskeletal adverse effects of ISO in AV patients. We did not find any significant difference in terms of serum vitamin D levels between the AE and C groups. In addition, there was no significant difference in frequency of ISO-induced adverse effects between the subgroups in AE group.

AV is a chronic inflammatory disease of the pilosebaceous unit. Although, its pathogenesis is not clearly understood, follicular hyperkeratinization, increased sebum production, propionibacterium acnes and inflammation have been reported to play a role^5,6^.

Recently, it is found that vitamin D deficiency is associated with many chronic diseases including cancers, cardiovascular diseases, metabolic syndrome, infectious and autoimmune skin diseases, also, AV^19,21,22^. It is considered that vitamin D is important not only in calcium homeostasis, but also in immune system regulation, cell growth and differentiation^23^. Vitamin D modulates the immune system and the proliferation and differentiation of the keratinocytes and sebocytes. Vitamin D is also known as anticomedogenic and antioxidant. The deficiency of vitamin D may facilitate the pathogenesis of AV^6,19^. The relationship between vitamin D and AV depends on possible anti-inflammatory effects of vitamin D^24^.

There are several studies to investigate relationship between serum levels of vitamin D and AV with inconsistent results, in the literature. There was no association between serum vitamin D level and AV in some of the studies^21–25^. Some studies, in contrast, revealed that significantly lower serum vitamin D level in acne patients compared to the healthy controls^26–28^. Yildizgören *et al*. evaluated a total of 43 patients with newly diagnosed nodulocystic acne and 46 healthy control subjects according to their 25 OH D levels^27^. The authors found that the patients with nodulocystic acne had significantly lower serum vitamin D levels compared with the control group. Moreover, a review and meta-analysis showed that lower serum vitamin D level and higher prevalence of vitamin D deficiency in patients with AV^28^. The findings of these studies suggest that there is a relationship between low vitamin D levels and AV. The two study groups also have AV and there was no significant difference in terms of the serum vitamin D levels between the study groups, in our study.

Recently, El-Hamd *et al*. performed a study including 90 patients with AV and age- and sex-matched 60 healthy controls. They found that the baseline serum 25 OH vitamin D levels were significantly lower in patients with AV than control group. The patients were treated with ISO dose of 0.75 mg/kg/day for a period of 3 months. After 3 months of ISO treatment, serum 25 OH vitamin D levels have increased significantly in patients with AV. The authors also found an inverse correlation between serum level of 25 OH vitamin D and severity of AV, before the treatment. They have reported that vitamin D may play a potential role in pathogenesis of the AV or AV may have a negative impact on vitamin D synthesis^19^. In contrast to this study, Toossi *et al*. found no significant difference in serum 25 OH vitamin D levels between AV patients and healthy controls. They also indicated no correlation between serum vitamin D levels and severity of AV^25^.

The effects of ISO on bone metabolism and bone physiology also have been investigated with several studies. ISO is a synthetic vitamin A analogue. Frankel *et al*. found that chronically vitamin A overdosed rats had increased osteoclastic activity, reduced osteoid formation, and reduced levels of 25 OH vitamin D^29^.

Miziolek *et al*. indicated that excessive intake of vitamin A may deteriorate functioning of vitamin D especially in subjects with a vitamin D deficiency (<50 nmol/l of 25 OH vitamin D), therefore a similar side effect may also appear in patients using ISO. The authors suggested that decreasing the use of ISO after bone injury or continuing the treatment at low dose with a concomitant correction of vitamin D and calcium status^30^. The vitamin D deficiency may be a condition predisposing to osteoporosis and decreased bone mineral density^31^. In fact, Boucher suggested that a measurement of serum 25 OH vitamin D and correction of vitamin D deficiency appear to be advisable before starting ISO treatment^32^. We did not find any correlation between serum vitamin D levels and cumulative ISO dose, ISO-induced musculoskeletal side effects. In our study, Group I had the lowest serum vitamin D level, but there was no significant difference between Group I, II and III in frequency of the musculoskeletal side effects. Therefore, according to our results, we are considering that the measurement or correction of 25 OH vitamin D deficiency is not necessary before ISO treatment for AV.

Ertugrul *et al*.^18^ investigated 35 females and 15 males with AV and measured 25 (OH) vitamin D, 1,25 dihydroxy vitamin D, and bone alkaline phosphatase, calcium, phosphate, and parathormone levels before and after 3 months of ISO treatment. They revealed that 1,25 dihydroxy vitamin D, parathormone, and bone alkaline phosphatase levels increased significantly after 3 months of ISO treatment. Hovewer, 25 OH vitamin D and calcium levels were significantly decreased after the treatment. They reported that ISO has an impact on vitamin D metabolism^18^. We found no correlation between serum vitamin D levels and cumulative ISO dose of the patients with AV.

There are limitations of this study. Since, our study is a cross-sectional study, it is important to support the relationship between the serum level of 25 OH vitamin D and ISO-induced musculoskeletal symptoms with larger patient groups and further prospective randomized controlled trials.

## Conclusion

We may conclude according to our results that, there is no relationship between the serum levels of 25 OH vitamin D and musculoskeletal side effects in AV patients. It is indicated that vitamin D deficiency have no effect on the musculoskeletal symptoms in patients receiving ISO for AV. Therefore, the measurement of serum 25 OH vitamin D level or intake of vitamin D supplements is not necessary before ISO treatment in patients with AV.

## Supplementary information

Supplementary information
